# Predictive validity of the SAD PERSONS and NO HOPE scales in a sample of suicide cases

**DOI:** 10.3389/fpsyg.2025.1554971

**Published:** 2025-04-24

**Authors:** Sergio Sanz-Gómez, Diego De La Vega-Sánchez, Adrián Alacreu-Crespo, Jorge Luis Ordoñez-Carrasco, María Isabel Perea-González, Aida Castell-Navarro, Julio Guija, Lucas Giner

**Affiliations:** ^1^Departamento de Psiquiatría de la Facultad de Medicina de la Universidad de Sevilla, Seville, Spain; ^2^Hospital Universitario Virgen Macarena, Seville, Spain; ^3^Departamento de Psicología y Sociología, Universidad de Zaragoza, Teruel, Spain; ^4^Institute of Legal Medicine of Sevilla, Seville, Spain

**Keywords:** suicide, SAD PERSONS scale, NO HOPE scale, predictive model, psychological autopsy

## Abstract

**Introduction:**

Suicide is a global public health issue necessitating evidence-based prevention strategies. Many individuals who die by suicide have had prior contact with healthcare services. Nearly half visit a primary care provider within a month before their death, and many visit emergency departments (EDs) frequently. Effective risk assessment in EDs is critical for suicide prevention but remains challenging. Inadequate risk assessments are a common error identified in suicide deaths. While clinical interviews are vital, risk assessment scales can support decision-making. The SAD PERSONS and NO HOPE scales are widely used but have limitations in predictive value.

**Materials and methods:**

A case–control study using psychological autopsy (PA) was conducted from 2006 to 2016. Data were collected from 662 individuals in southern Spain, including 487 suicide cases and 175 controls. PAs involved interviews with close relatives and were conducted by trained psychiatrists or psychologists. The SAD PERSONS and NO HOPE scales were utilised, and data were analysed using sensitivity, specificity, and logistic regression to develop an improved predictive model.

**Results:**

The SAD PERSONS scale showed high specificity but low sensitivity in predicting suicide risk. In the non-suicide group, 91.6% were classified as low risk. In the suicide group, nearly half were classified as low risk (49.6%). The modified SAD PERSONS scale showed similar results. The NO HOPE scale had low sensitivity but high specificity. An improved predictive model incorporating key variables from both scales demonstrated higher sensitivity (93.609%) and specificity (91.608%).

**Discussion:**

The SAD PERSONS scale has limitations in effectively predicting suicide risk, particularly due to its focus on non-modifiable factors. Adding variables from the NO HOPE scale improves predictive utility. Comprehensive clinical assessments, considering psychological, social, and environmental factors, are essential for accurate suicide risk evaluation and tailored intervention.

## Introduction

People who die by suicide contact the healthcare system often, either through a primary care physician or an emergency department: nearly half of suicide completers visited a primary care provider 1 month before their death, and three out of four made a visit in the previous year (Luoma et al., 2002). Individuals who present to emergency departments (EDs) with suicidal behaviour are often chronic users of EDs and are at increased risk for either repeated self-harm or death by suicide (Ceniti et al., 2020). Therefore, risk assessment of suicidality in the ED is also of paramount importance for suicide prevention. Nevertheless, suicide risk assessment is challenging. A recent review analysing healthcare system errors in suicide deaths identified inadequate and/or incomplete risk assessment as a common error, sometimes exacerbated by lack of family involvement and poor information flow between healthcare settings (Wyder et al., 2020).

Appropriate and evidence-based risk assessment is crucial for suicide prevention in healthcare. While clinical interviews are central to assessing suicidal behaviour, additional tools can support decision-making (Meyer et al., 2010). However, identifying a reliable assessment tool is challenging. A recent meta-analysis found no single instrument with sufficient diagnostic accuracy or adequate data for accurate analysis (Runeson et al., 2017). Is for this reason, that is crucial to have scales with good predictive value of suicide.

One of this scales, the SAD PERSONS scale is widely used as suicide assessment tool (Ayuso-Mateos et al., 2012; Bolton et al., 2015; Saab et al., 2022). It is an acronym utilised as a mnemonic device. Scoring is calculated on the basis of 10 items: S (male sex), A (age younger than 19 or older than 45 years), D (depression), P (previous attempts), E (alcohol or substance use), R (rational thinking loss), S (lack of social support), O (organised plan), N (no spouse/partner), S (poor physical health). In the Modified SAD PERSONS, several items (Depression or Hopelessness, Loss of Rational Thinking, Organised or Serious Attempt, and Stated Future Intent) score double (Warden et al., 2014).

The SAD PERSONS has been heavily criticised for its lack of predictive value with regard to both suicide and suicide attempts (Bolton et al., 2015). A recent meta-analysis highlights its excellent specificity as a key feature (97%; CI 96–98) as opposed to its limited sensitivity (15%; 95% CI 8–24; Runeson et al., 2017). In a systematic review that evaluated the use of the SAD PERSONS in predicting suicide outcomes none of the three studies examined showed that the scale accurately predicted suicidal behaviour (Warden et al., 2014). Despite its lack of support, it is a popular tool in clinical practice. This is mainly due to its short length and mnemonic rule, which makes it easy to learn and apply (Ministerio De Sanidad, P. S. E. I, 2012). A review of suicide risk assessment in the United Kingdom found that the SAD PERSONS was the most widely used tool in healthcare settings (Fedorowicz et al., 2023). Given the lack of evidence to support its use, no clinical guidelines include it in their risk assessment scales. However, it is recommended in some guidelines and expert documents as a reminder of the risk factors that should be assessed for suicide (Ayuso-Mateos et al., 2012; Wasserman, 2021).

Another scale, the NO HOPE, is a scale developed by Shea (1999). It consists of six items, the initials of which form the name of the instrument: N (no support), O (organised plan), H (hopelessness), O (ongoing medical illness), P (previous attempts), E (excessive substance use). It is intended to provide a more thorough assessment of suicide risk, including the concept of hopelessness (Wasserman, 2021). Notwithstanding the extensive utilisation of these scales, there is an absence of scientific literature that provides empirical validation of their application, nor the concurrent utilisation of the SAD PERSONS and NO HOPE scales.

## Objectives

The objectives of this study were to assess the predictive power of the SAD PERSONS and NO HOPE scales on death by suicide and to develop an improved predictive model of death by suicide based on the items of the SAD PERSONS and NO HOPE scales. We hypothesise that the lack of predictability of the scales is due not so much to a failure in the selection of items in the scales, but to the relative weight given to each item.

## Methods

### Design

A case–control study was carried out in the province of Seville, Spain, between 2006 and 2016 using the psychological autopsy (PA) method, a validated and widely recommended approach for the study of suicide. This methodology involves collecting retrospective information about the deceased through structured interviews with relatives or close contacts. Interviews were typically conducted between 3 and 18 months after the death, most often in person at the Department of Psychiatry of the University of Seville. In exceptional cases, interviews were carried out at the interviewee’s home or workplace, depending on their preference.

Interviews were carried out by a team consisting of two psychiatrists and two psychologists, all trained in psychological autopsy procedures by the principal investigator. After each interview, the data were reviewed in an interdisciplinary consensus meeting to reach a diagnostic conclusion.

The autopsy protocol gathered a wide range of variables, including psychiatric history, presence of mental disorders, suicidal ideation or attempts, substance use, medical conditions, stressful life events, interpersonal relationships, social support, and contextual risk and protective factors. The protocol used is based on previously published models (Hawton et al., 1998; de la Vega Sánchez et al., 2020) and is available upon request from the corresponding author.

All procedures were conducted in accordance with the ethical standards of the relevant institutional and national research committees, and with the 1975 Declaration of Helsinki, as revised in 2013. The study was approved by the Ethics Committee of the University of Seville (approval number: 5012008), and all participants signed written informed consent prior to their participation.

### Participants

The sample included 662 individuals from the province of Seville, Spain, between 2006 and 2018, comprising 487 suicide cases and 175 controls who died from other sudden causes (natural and accidental deaths). According to the National Statistics Institute (2022), the total population of the province of Seville in 2024 was 1,967,746, with a suicide rate of 8.28 per 100,000 inhabitants.

The cause of death and its classification as suicide or non-suicide was determined by forensic physicians following a forensic investigation and mandatory legal autopsy at the Institute of Legal Medicine in Seville. Deaths occurring in prison or under police custody were excluded from the study. The suicide group included individuals over the age of 18 residing in the province of Seville, whose death was certified as suicide and whose relatives provided informed consent to participate in the interview. The control group consisted of individuals over the age of 18 from the same geographical area, whose death was certified as sudden or accidental (excluding suicide), and whose relatives also consented to the interview.

At the Institute of Legal Medicine in Seville, both relatives and close contacts of the suicide cases and of the controls were invited to participate in the study. While all families were approached under the same protocol, participation rates were higher among relatives of individuals who died by suicide.

### Measures

The psychological autopsy protocol followed previously published procedures (Hawton et al., 1998) and is described in detail elsewhere (de la Vega Sánchez et al., 2020; Giner et al., 2013). This psychological autopsy procedure included the SAD PERSONS, a scale originally developed by Patterson et al. (1983) and the NO HOPE scales (Shea, 1999). Each item assesses the absence or presence of a risk factor for suicidality with a score of 0 or 1 for a total score ranging from 0 to 10. A score equal to or less than 4 indicates “low suicide risk,” between 5 and 6 “moderate suicide risk,” and between 7 and 10 “high risk.” In the Modified SAD PERSONS scale four specific items are scored with double weight: depression or hopelessness, rational thinking loss, organised or serious attempt, and sickness (stated future intent). These weighted items are marked with an asterisk in Table 1. In this version, total scores range from 0 to 14, with a score equal to or less than 5 indicates low suicide risk, between 6 and 8 moderate suicide risk, and between 9 and 14 high risk (Warden et al., 2014). In the NO HOPE, each item is scored dichotomously for a total score ranging from 0 to 6 (Shea, 1999). There are no proposed cut-off points for suicide risk classification.

**Table 1:** Modified SAD PERSONS and NO HOPE Scales.

SAD PERSONS	Score	NO HOPE	Score
Sex: male	1	No framework or meaning	1
Age: <19 or >45 years	1	Overt change in clinical condition	1
Depression or hopelessness	1 (2)*	Hostile interpersonal environment	1
Previous attempts or psychiatric care	1	Out of hospital recently	1
Excessive alcohol or drug use	1	Predisposing personality factors	1
Rational thinking loss	1 (2)*	Excuses for dying are present and strongly believed	1
Separated/divorced/widowed	1		
Organised or serious attempt	1 (2)*		
No social support	1		
Sickness (Stated future intent)	1 (2)*		

*Modified SAD PERSONS.

### Data analysis

In an initial analysis of the SAD PERSONS predictive model, we classified the sample of suicides and controls according to the risk classifications proposed in the SAD PERSONS and the modified SAD PERSONS (low, moderate, high). We then measured the sensitivity, specificity, and the area under the ROC curve of the scale for classifying suicide and control samples. In the NO HOPE predictive analysis, given the absence of established cut-off points, we calculated sensitivity and specificity for two points, namely 2 and 5.

Subsequently, in the development of an improved predictive model, we developed a forward regression model using the variables from the SAD PERSONS and NO HOPE scales. We utilised the AllSetReg tool (Domenech et al., 2013), SPSS script, to select the best model. Sensitivity, specificity, and area under the ROC curve were calculated for each model. Subsequently, the variables of the selected model were entered into a logistic regression to determine the variable coefficients and the logistic regression equation.

## Results

### SAD PERSONS predictive analysis

Contingency analysis was used to examine SAD PERSONS scores by type of death (suicide vs. non-suicide). The results are shown in Table 2.

**Table 2:** Contingency table of original SAD PERSONS score by type of death.

	Group	Low risk	Medium risk	High risk	Total
Non-suicide	Count	131	11	1	143
	% of within control	91.6%	7.7%	0.7%	100%
Suicide	Count	132	108	26	266
	% of within suicide	49.6%	40.6%	9.8%	100%
Total	Count	263	119	27	409
	% of within SAD PERSONS	64.3%	29.1%	6.6%	100%

Considering those scoring as medium or high risk as positive, the SAD PERSONS scale had a sensitivity of 50.376 (95% IC = 44.405–56.336), a specificity of 91.608 (95% IC = 85.905–95.135) and an area of the ROC curve of 0.802 (95% CI 0.758–0.845).

The results show that in the control group, 91.6% were classified as low risk on the SAD PERSONS scale, while in the suicide group, 49.6% were classified as low risk, 40.6% as moderate risk, and 9.8% as high risk. Using the modified SAD PERSONS, 97.2% of the control group scored as low risk. In the suicide group, 47.0% were classified as low risk and 10.4% as high risk.

### Modified SAD PERSONS predictive analysis

Contingency analysis was performed to examine Modified SAD PERSONS scores by type of death (suicide vs. non-suicide). The results are shown in Table 3.

**Table 3:** Contingency table of modified SAD PERSONS score by type of death.

	Group	Low risk	Medium risk	High risk	Total
Non-suicide	Count	140	3	1	144
	Expected frequency	93.0	40.9	10.1	144
	% of within control	97,2%	2,1%	0,7%	100%
Suicide	Count	126	114	28	268
	Expected frequency	173	76.1	18.9	268
	% of within suicide	47%	42.5%	10,4%	100%
Total	Count	266	117	29	412
	Expected frequency	266	117	29	412
	% of within SAD PERSONS	64.6%	28.4%	7.0%	100%

Considering those scoring medium or high risk as positive, the modified SAD PERSONS scale had a sensitivity of 52.985 (95% IC = 47.01.-58.876), a specificity of 97.222 (95% IC = 93.076–98.915) and an area of the ROC curve of 0.910 (95% CI 0.881–0.030).

### NO HOPE predictive analysis

Using cut-off point 2, the sensitivity was 11.654 (95% CI = 8.333–16.067) and the specificity was 98.601 (95% CI = 95.044–99.616). Using the cut-off point 5, the sensitivity was 0.376 (95% CI = 0.066–2.098) and the specificity was 100 (95% CI = 97.384–100). The scale had a ROC area of 0.834 (95% CI 0.791–0.877).

### Development of an improved predictive model

The model recommended by AllSetReg included 10 variables: male sex, age, depression, previous attempts, accessible method, physical illness, sense of purpose, clinical status, predisposing personality, and reasons for maintaining dying. The model including these variables had a sensitivity of 93.609%, specificity of 91.608%, ROC curve area of 0.968, and −2LL (likelihood) value of 175.298.

The selected variables were entered into logistic regression to determine the coefficients of each variable and the resulting logistic regression equation (Table 4).

**Table 4:** Default values and values in the new logistic regression model.

SAD PERSONS	Original	Modified	B value in the proposed model	Exp(B)
Sex (male)	1	1	1,729	5,632
Age (<19 or >45)	1	1	0,667	1,948
Depression (yes)	1	2	1,881	6,560
Previous (suicide attempts)	1	1	2,341	10,389
Ethanol (alcohol abuse)	1	1		
Rational (loss of rational thinking)	1	2		
Single (single, separated, or widowed)	1	1		
Organised (plan for suicide)	1	2	4,569	96,424
No social (lack of social support)	1	1		
Sickness (Stated future intent)	1	2	−2,042	0,130
*NO HOPE*				
No meaning	1		2,147	8,558
Overt change in clinical condition	1		1,826	6,209
Hostile interpersonal environment	1			
Out of hospital recently	1			
Predisposing personality factors	1		1,385	3,994
Excuses (reasons for dying)	1		2,893	18,048

## Discussion

The results reveal the SAD PERSONS scale’s ineffectiveness in predicting suicide. While the scale shows high specificity in the non-suicide group, correctly identifying 91.6% as low risk, it fails in sensitivity for the suicide group, with almost half classified as low risk. This indicates the scale’s poor performance in detecting high-risk cases. The modified SAD PERSONS showed minimal improvement in specificity and similarly low sensitivity. Thus, neither version satisfactorily predicts suicidal intent, though both have excellent specificity. Adding NO HOPE items (reasons for dying, lack of meaning, change in clinical situation, and predisposing personality traits) could enhance predictive utility. These results are consistent with other studies that have examined the predictive value of both the SAD PERSONS and its modified version for recurrent self-harm (Bolton et al., 2012; Katz et al., 2017; Wu et al., 2014).

### SAD PERSONS reach and consequences

If the main interest of suicide risk scales is to discriminate whether a patient will make a lethal suicide attempt or not, this crucial aspect is something that the SAD PERSONS scale cannot do. In addition, the SAD PERSONS assesses some non-modifiable factors, such as gender and age, so its widespread use poses further problems. Women between the ages of 19 and 45 consistently appear to have a lower risk of suicide than younger or older men. Thus, the widespread use of this scale allows mental health professionals not only to perpetuate the myth that suicide attempts or non-suicidal self-injury in young women are often attention-seeking behaviour, but also to act on this myth and make unwise treatment decisions.

It is crucial to recognise that simply ticking off a checklist of risk factors is insufficient for predicting suicide. A recent meta-analysis spanning 50 years of research on suicide behaviour risk factors underscored this point, revealing that our current ability to predict suicide outcomes only marginally surpasses chance levels across various analyses (Franklin et al., 2017). The study highlighted a significant gap in suicide research—examination of the combined impact of multiple risk factors—which undermines effective prevention strategies. Therefore, a thorough assessment conducted by an expert clinician is essential to accurately evaluate suicide risk, moving beyond reliance on single scales or disparate risk factor assessments.

According to the NICE guidelines, risk assessment scales should not replace clinical interviews when assessing suicide risk. In fact, psychosocial risk assessment is associated with better health outcomes [National Collaboration Centre for Mental Health (UK), 2012]. Therefore, it is critical that mental health professionals take the time to conduct thorough interviews and gather as much information as possible before making decisions about a patient’s suicide risk.

### Evidence-based assessment of suicide risk

According to our results, non-modifiable factors (gender, age, and predisposing personality traits) were the least associated with death by suicide. In fact, age did not even differentiate between the groups. Among the relevant factors were presence of reasons for dying, lack of purpose in life, history of suicide attempts and, most importantly, accessibility to a method of suicide. The fact that physical illness appears as a protective factor for death by suicide is counterintuitive and contradicts previous literature (Giner et al., 2013). This may be due to bias in the control group sampling, which stem from the requirement for coroner’s autopsies, encompassing sudden deaths due not only to accidents but also to physical illnesses like chronic heart conditions. This bias could skew towards conditions such as coronary heart disease. Similarly, alcoholism, linked to various chronic diseases and suicide, may introduce a similar bias.

Access to means of suicide is a critical factor in suicide prevention, reflected in various mnemonic rules like PIMP (means), PLAID (access to means), SLAP (availability), and SIMPLE STEPS (method; McGlothlin et al., 2016). According to the interpersonal theory of suicide, familiarity and personal experience with suicide methods over a lifetime can increase the ability to harm oneself (Joiner, 2012). Research on lifetime exposure to firearms supports this, linking it directly to suicide risk (Anestis and Capron, 2018). Thus, restricting access to potentially harmful methods is crucial not only for immediate crisis intervention, but also for long-term suicide prevention efforts.

Reasons for dying and lack of purpose in life are intertwined and complex concepts representing cognitive and affective components of life experience. The former involves beliefs, values, and motivations providing purpose, while the latter signifies feelings of emptiness, hopelessness, and lack of meaning, often associated with depressive disorder or psychological distress (Conejero et al., 2018). Our findings stress the importance of assessing these factors during ED triage, but their presence alone does not suffice for comprehensive risk evaluation. Given their complexity and links to various mental health conditions, a thorough assessment by a trained professional is crucial for accurate understanding of suicidality and mental well-being. While our model includes several key psychological and contextual factors, future research should expand upon this by incorporating dynamic and modifiable variables, such as acute psychological distress, recent life events, and fluctuations in suicidal ideation. These aspects may capture short-term risk more accurately and improve the temporal sensitivity of suicide risk prediction tools.

Instruments with higher sensitivity and specificity often include items related to suicidal ideation, previous attempts, access to lethal means, psychiatric history, and social support. Despite this, recent reviews indicate wide variability in the sensitivity and specificity of commonly used scales, ranging from 1 to 50% for both parameters (Saab et al., 2022). These discrepancies may stem from methodological differences and highlight limitations in current scale formats for assessing suicide risk. Currently, clinician assessment remains the most suitable method for nuanced suicide risk evaluation.

While the logistic regression model developed in this study shows high sensitivity and specificity, its application in clinical settings may be limited by the need to calculate probabilities based on multiple variables. Future research should aim to validate this model through prospective studies conducted in real-world clinical environments, such as emergency departments or primary care. Testing the model’s predictive performance in routine practice would provide valuable evidence on its clinical utility and feasibility, and help determine whether its integration could improve suicide prevention outcomes. To enhance its practical utility, future research could focus on developing a simple digital tool—such as an app or web-based calculator—that allows clinicians to input patient data and automatically obtain a risk estimate. This could help bridge the gap between statistical modelling and real-world clinical decision-making.

In summary, while our findings highlight the limitations of commonly used scales and support the need for comprehensive clinical assessment, they also open a promising avenue for innovation: integrating validated predictive models into user-friendly digital tools that can enhance clinical decision-making. This perspective sets the stage for future work and informs the conclusions that follow.

### Strengths and limitations

Strengths of the study include the use of a case–control design with psychological autopsy, which allowed a detailed examination of the risk factors and circumstances of death by suicide compared with controls. The study also involved a multidisciplinary approach, with professionals from psychiatry and psychology contributing to the assessment and analysis process.

However, there are limitations. First, the control group may be biased, as individuals who died of non-suicidal sudden death may have a higher likelihood of alcohol consumption and chronic disease, which could potentially confound the comparison with the suicide cases. Second, the study relied on retrospective data collected through psychological autopsies, which introduces the possibility of recall bias or incomplete information. Despite efforts to include multiple informants and conduct consensus meetings to enhance reliability, subjective memory distortions may still have influenced the accuracy of the reported data. Third, interviews were conducted between 3 and 18 months after death—an extended timeframe that exceeds the commonly recommended window of 2 to 6 months. This extended timeframe may have affected the accuracy of some recollections and, consequently, the sensitivity and specificity of the predictive models. Finally, the study was conducted in a single geographic region—southern Spain—which may limit the generalizability of the findings to other populations with different cultural, social, or healthcare contexts. Future research should aim to validate these results in diverse settings and through prospective designs.

These limitations should be considered when interpreting the results and generalising them to broader populations.

## Conclusion

The SAD PERSONS scale has limitations in providing a comprehensive assessment of suicide risk. The scale focuses on non-modifiable factors such as gender and age, which may overlook important indicators of suicidality in certain populations, such as young women. It fails to capture the complexity and multidimensionality of suicide risk, highlighting the need for a more comprehensive assessment. However, and more importantly, as shown in this and other studies with other types of samples (in the context not only of suicide deaths but also of suicide attempts and suicidal ideation…), the scale does not meet the minimum standards of sensitivity and specificity that make it relevant. Clinical guidelines should reflect this issue and emphasise that the use of such instruments is for screening purposes only.

These problems are shared by other scales used to triage suicidality, such as the NO HOPE. Individual risk factors alone are not sufficient for accurate prediction. Suicide risk assessment requires consideration of a range of factors beyond the items included in the SAD PERSONS scale. Factors such as reasons for dying, lack of purpose in life, previous suicide attempts, and access to means of suicide may play a critical role in understanding suicidality, but they cannot be adequately assessed by a single scale or checklist. To effectively assess suicide risk, a comprehensive clinical assessment conducted by a trained professional is essential. This assessment should consider a variety of factors, including psychological, social, and environmental, to gain a comprehensive understanding of the individual’s unique circumstances and the presence of underlying risk factors. This approach allows for a more accurate assessment and provides the opportunity for tailored intervention and support.

## Data Availability

The raw data supporting the conclusions of this article will be made available by the authors, without undue reservation.
